# Monitoring cancer prognosis, diagnosis and treatment efficacy using metabolomics and lipidomics

**DOI:** 10.1007/s11306-016-1093-7

**Published:** 2016-08-16

**Authors:** Emily G. Armitage, Andrew D. Southam

**Affiliations:** 1 Centre for Metabolomics and Bioanalysis (CEMBIO), Faculty of Pharmacy, Universidad CEU San Pablo, Campus Monteprincipe, Boadilla del Monte, 28668 Madrid, Spain; 2 Wellcome Trust Centre for Molecular Parasitology, Institute of Infection, Immunity and Inflammation, College of Medical Veterinary and Life Sciences, University of Glasgow, Glasgow, G12 8TA UK; 3 Glasgow Polyomics, Wolfson Wohl Cancer Research Centre, College of Medical Veterinary and Life Sciences, University of Glasgow, Glasgow, G61 1QH UK; 4 School of Biosciences, University of Birmingham, Edgbaston, Birmingham, B15 2TT UK

**Keywords:** Mass spectrometry, Nuclear magnetic resonance, Leukemia, Stratified medicine, Nutraceutical, Drug redeployment

## Abstract

**Introduction:**

Cellular metabolism is altered during cancer initiation and progression, which allows cancer cells to increase anabolic synthesis, avoid apoptosis and adapt to low nutrient and oxygen availability. The metabolic nature of cancer enables patient cancer status to be monitored by metabolomics and lipidomics. Additionally, monitoring metabolic status of patients or biological models can be used to greater understand the action of anticancer therapeutics.

**Objectives:**

Discuss how metabolomics and lipidomics can be used to (i) identify metabolic biomarkers of cancer and (ii) understand the mechanism-of-action of anticancer therapies. Discuss considerations that can maximize the clinical value of metabolic cancer biomarkers including case–control, prognostic and longitudinal study designs.

**Methods:**

A literature search of the current relevant primary research was performed.

**Results:**

Metabolomics and lipidomics can identify metabolic signatures that associate with cancer diagnosis, prognosis and disease progression. Discriminatory metabolites were most commonly linked to lipid or energy metabolism. Case–control studies outnumbered prognostic and longitudinal approaches. Prognostic studies were able to correlate metabolic features with future cancer risk, whereas longitudinal studies were most effective for studying cancer progression. Metabolomics and lipidomics can help to understand the mechanism-of-action of anticancer therapeutics and mechanisms of drug resistance.

**Conclusion:**

Metabolomics and lipidomics can be used to identify biomarkers associated with cancer and to better understand anticancer therapies.

## Introduction: cancer metabolism

Cancer initiation and progression is associated with specific changes to cellular metabolism that are not simply by-products of the disease; instead they appear to drive the disease (Boroughs and DeBerardinis 2015; Wishart 2015). Activated oncoproteins alter cell metabolism (Kimmelman 2015; Sancho et al. 2015) and some metabolic enzymes are now being considered as oncoproteins (Migita et al. 2009). At the genetic level cancer is very complex and heterogeneous (Lohr et al. 2014), however at the metabolic level only a few processes are altered (Wishart 2015). The identification and measurement of cancer-specific metabolic and lipid markers from low-invasive patient samples has the potential to monitor prognosis and disease in cancer patients.

**Fig. 1:**
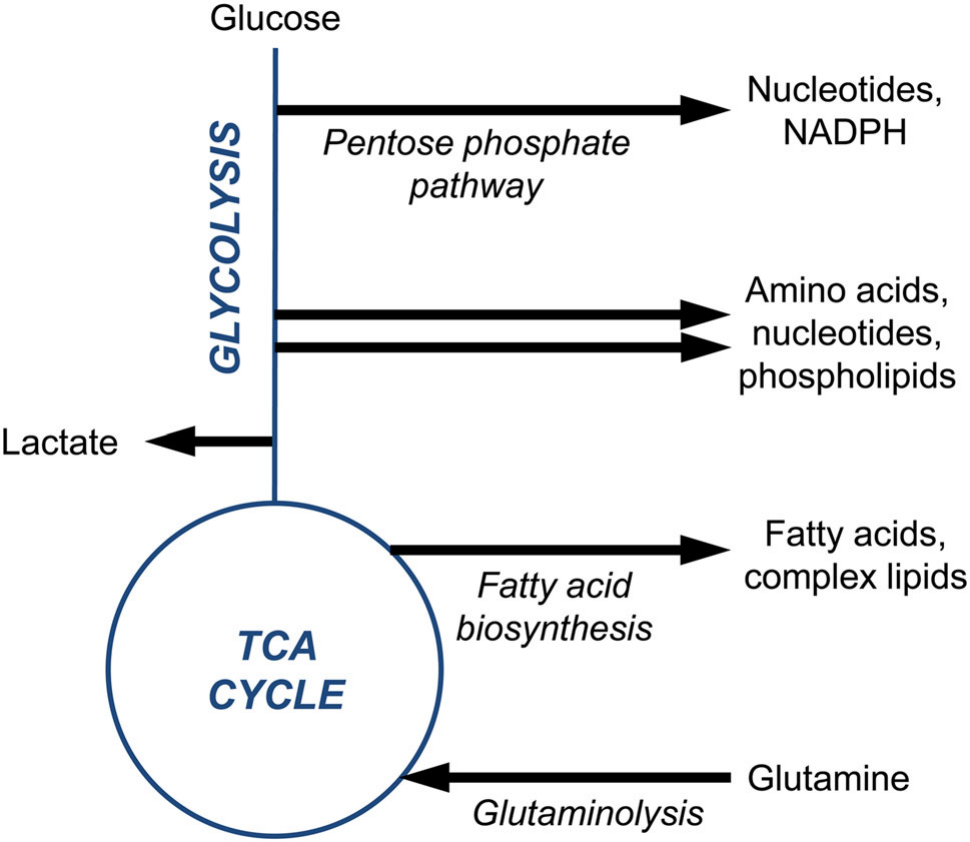
A simplified overview of metabolic changes that occur in cancer. Cancers often exhibit increased aerobic glycolysis resulting in glucose carbon being directed towards lactate and the anabolic synthesis of nucleotides, amino acids and lipids. This is associated with disruption of the TCA cycle and the increased use of glutamine as a carbon source (glutaminolysis). Cancer-induced increase of the pentose phosphate pathway can increase NADPH recycling to protect cells against oxidative stress

For metabolic or lipid markers to be robust indicators of cancer they need to be anchored in biochemical knowledge of tumour metabolism (Fig. 1). High glucose demand and aerobic glycolysis are common metabolic traits of cancer cells (Vander Heiden et al. (2009); Warburg 1956). This is often accompanied by mutagenic disruption to TCA cycle enzymes (King et al. 2006), creating a metabolic phenotype that directs glucose carbon towards anabolic synthesis (Boroughs and DeBerardinis 2015). Additionally, this favours NADPH recycling to maintain glutathione levels and an optimal cellular redox status (Patra and Hay 2014) (Fig. 1). Functional mitochondria are essential to cancer cells with TCA cycle disruption (Wallace 2012). Mitochondria contribute towards anabolic biosynthesis in tumours (Ahn and Metallo 2015), including de novo fatty acid biosynthesis—a process that is upregulated in several cancers (Currie et al. 2013). Glutaminolysis is a key metabolic process in MYC driven cancers whereby carbon from the catabolism of glutamine is imported into the mitochondria to maintain mitochondrial membrane potential (Wise and Thompson 2010). Carbon from glutamine is also used for the anabolic synthesis of proteins and nucleotides (DeBerardinis et al. 2007). Additionally, the local tumour environment plays a key role in the metabolism of cancer cells. Here, nutrient- and oxygen-poor tumour cells scavenge alternate carbon sources—lactate (Doherty and Cleveland 2013), acetate (Kamphorst et al. 2014; Schug et al. 2015) and lipids (Kamphorst et al. 2013)—to maintain energy production and anabolic synthesis.

This review will discuss considerations required for metabolic monitoring of cancer patients in the clinic. Current examples of its application to monitor disease risk and incidence, disease staging and understand the mechanism-of-action of anticancer therapeutics [pharmacometabolomics (Kaddurah-Daouk et al. 2008; Lindon et al. 2006)] will be discussed.

## Metabolomic and lipidomic strategies

### Sample types

The identification of metabolic markers that can clinically monitor cancer status requires access to patient samples (Mayers et al. 2014). Analysis of tissue samples provides mechanistic understanding of cancer (Ren et al. 2016; Rocha et al. 2015; Wang et al. 2013), however such invasive samples are less suited for regular patient monitoring. An exception is magnetic resonance imaging (MRI) where metabolic profiles of tumour tissue can be non-invasively obtained (Gill et al. 2014). Low-invasive patient samples (e.g. serum, urine) are ideal for regular patient monitoring as they offer minimal patient discomfort and can justifiably be taken from healthy (control) patients. The location of the cancer may influence the chosen sample type, e.g. urine for bladder cancer (Jin et al. 2014), breath for lung cancer (Li et al. 2015). Low invasive samples are generally extracellular fluids. Here, the metabolic profile is dependent on cellular uptake and excretion from all bodily processes (not just the cancer), which must be considered during data interpretation. Pharmacometabolomics analysis to understand the mechanism-of-action of anticancer therapeutics often begins in cell lines (Southam et al. 2015), before progressing to ex vivo studies (Koczula et al. 2016) and then patient samples (Schuler et al. 2015).

### Analytical approaches

The study of cancer metabolism is most typically done by steady-state metabolomics or lipidomics using liquid chromatography-mass spectrometry (LC–MS) (Mayers et al. 2014; Kuhn et al. 2016; Piszcz et al. 2016), gas chromatography-MS (GC–MS) (Xie et al. 2015; Wittmann et al. 2014), direct infusion MS (DIMS) (Southam et al. 2015; Li et al. 2013; Southam et al. 2007) or nuclear magnetic resonance (NMR) spectroscopy (Fages et al. 2015; Lodi et al. 2013). To retain spatial information, metabolic imaging approaches can be used [e.g. matrix-assisted laser desorption/ionization MS (Krasny et al. 2015)]. Analytical techniques more suited to the clinical setting are emerging, including liquid extraction surface analysis (LESA) MS to profile lipids directly from dried blood spots (Griffiths et al. 2015); portable hand-held Raman spectrometery (Mabbott et al. 2013); and rapid evaporative mass spectrometry (Schaefer et al. 2009). The intelligent knife (iKnife) where a surgical scalpel is coupled to a MS detector to measure intraoperative real-time lipidomics capable of distinguishing tumour tissue from healthy tissue is an example for the latter (Balog et al. 2013).

### Stable isotopic labelling analysis

Stable isotope-labelled compounds (that contain ^13^C, ^2^H, ^15^N atoms; most commonly ^13^C-glucose and ^13^C-glutamine) can be traced into metabolites and lipids using MS or NMR metabolomics and lipidomics. This provides dynamic pathway information, which can inform on cancer processes (Kamphorst et al. 2013) and the mechanism-of-action of anticancer drugs (Southam et al. 2015). The non-toxic nature of stable isotopes enables their use in patients, including to demonstrate the heterogeneous metabolic nature of lung cancers (Hensley et al. 2016). Furthermore, stable-isotope labelling combined with hyperpolarized MRI metabolomic imaging can monitor cancer stages and therapy response in vivo by measuring the conversion of hyperpolarized ^13^C glucose or ^13^C-pyruvate to ^13^C-lactate in tumours (Rodrigues et al. 2014; Saito et al. 2015). The large concentration differences of lactate between tumour and healthy tissue allow for more sensitive and precise tumour detection than ^18^fluoro-2-deoxyglucose positron emission tomography (^18^FDG-PET) where tumour and surrounding tissue ^18^FDG levels can sometimes show poor contrast (Rodrigues et al. 2014).

## Identification of metabolic cancer biomarkers

### Maximising the clinical value of metabolic cancer biomarkers

Several metabolic markers have been associated with cancer status (Table 1), however this information is yet to be used for routine cancer screening in the clinic. To ensure future success of metabolic biomarkers in cancer patients certain aspects must be considered. Biomarkers should be more informative, less invasive and/or cheaper than current approaches (e.g. histology). To identify specific metabolic biomarkers of cancer, patient-to-patient variation—including ethnicity, sex, nutritional status, general health—should be minimised. The sampling procedure must be technically reproducible and the study size large enough to provide adequate statistical power. Different genetic mutations create subtle differences in metabolism, for instance RAS transformation will increase cellular glucose uptake and use in anabolic processes (Boroughs and DeBerardinis 2015). Therefore, genetic phenotyping of patient cancers [e.g. RAS status (Bertini et al. 2012)] would aid data interpretation and allow biomarkers to be assigned to specific mutations. Spectral features identified as biomarkers should be fully annotated and anchored in a sound biochemical understanding of cancer. This includes distinguishing metabolic cancer traits from general whole body metabolism and the metabolism of therapeutic drugs. Collections or ‘panels’ of markers are generally favoured over single biomarkers (Zang et al. 2014) as they better describe multifactorial metabolic processes. Furthermore, to overcome inter-patient variation of baseline metabolite levels, biomarkers can be measured as ratios of pairs/groups of compounds rather absolute intensity measurements of individual compounds (Zeng et al. 2015).

**Table 1:** Recent metabolomics and lipidomics studies indicating biomarkers of cancer risk, diagnosis, prognosis, remission or relapse

Cancer type	Sample type and study size	Key metabolic observations	Metabolic pathway(s) affected	Analytical platform(s)	Study design	Reference
*(A) Metabolic makers of cancer:*						
Bladder cancer	Urine Cohort 1: Cancer *n* = *66* Healthy control *n* = *266* Cohort 2: Cancer *n* = *29* Healthy control *n* = *79*	Increased in cancer: palmitoyl sphingomyelin, lactate Decreased in cancer: adenosine, succinate	Energy metabolism Lipid metabolism Nucleotide metabolism	LC–MS GC–MS	Case–control	(Wittmann et al. 2014)
Bladder cancer	Urine Cancer *n* = *138* Healthy control *n* = *121*	Increased in cancer: succinate, pyruvate, oxoglutarate, carnitine, phosphoenolpyruvate, trimethyllysine, isovalerylcarnitine, octenoylcarnitine, acetyl-CoA Decreased in cancer: melatonin, glutarylcarnitine, decanoylcarnitine	Energy metabolism Fatty acid β-oxidation	LC–MS	Case–control	(Jin et al. 2014)
Breast cancer	Plasma Cancer *n* = *5* Benign case *n* = *6* Healthy control *n* = *9*	Increased in cancer: phosphatidylglycerol (36:3), glucosylceramide (d18:1/15:1) Decreased in cancer: phosphatidylinositiols (PI): PI(16:0/16:1), PI(18.0/20.4) and PI(16:0/18:1)	Lipid metabolism Phospholipid metabolism	LC–MS	Case–control	(Yang et al. 2015)
Breast cancer	Plasma Cohort 1: Cancer *n* = *35* Healthy control *n* = *35* Cohort 2: Cancer *n* = *103* Healthy control *n* = *41* Serum Cohort 1: Cancer *n* = *103* Healthy control *n* = *31* Cohort 2: Cancer *n* = *80* Healthy control *n* = *70*	Decreased in cancer: Aspartate	Amino acid metabolism	LC–MS GC–MS	Case–control	(Xie et al. 2015)
Chronic lymphocytic leukaemia	Serum Indolent *n* = *51* Aggressive *n* = *42* Healthy control *n* = *45*	Increased in aggressive disease compared to indolent disease: Acetylcarnitine, acylcarnitines	Fatty acid β-oxidation	LC–MS	Case–control	(Piszcz et al. 2016)
Colorectal cancer	Serum Cancer *n* = *52* Healthy control *n* = *52*	Increased in cancer: lysophosphatidylcholines (LPC): LPC(16:0), LPC(18:2), LPC(20:4) and LPC(22:6) Decreased in cancer: palmitic amide, oleamide, hexadecanedioic acid, octadecanoic acid, eicosatrienoic acid, myristic acid	Lipid metabolism Phospholipid metabolism	DIMS	Case–control	(Li et al. 2013)
Gastric adenocarcinoma	Urine Cancer *n* = *43* Benign case *n* = *40* Healthy control *n* = *40*	Increased in cancer: Alanine, 3-indoxylsulfate Decreased in cancer: 2-hydroxyisobutyrate	Amino acid metabolism Oxidative stress	NMR	Case–control	(Chan et al. 2016)
Leptomeningeal carcinomatosis	Cerebrospinal fluid Cancer *n* = *26* Healthy control *n* = *41*	Increased in cancer: Alanine, citrate, lactate Decreased in cancer: Creatine, myo-inositol	Amino acid metabolism Energy metabolism Lipid metabolism	NMR	Case–control	(An et al. 2015)
Lung cancer	Serum Cohort 1: Cancer *n* = *23* Healthy control *n* = *23* Cohort 2: Cancer *n* = *9* Healthy control *n* = *9*	Increased in cancer: fatty acid amide, lysophosphatidylcholines Decreased in cancer: Free fatty acids	Lipid metabolism Phospholipid metabolism	LC–MS	Case–control	(Li et al. 2014)
Lung cancer	Breath Cancer *n* = *85* Benign case *n* = *34* Healthy control *n* = *85*	Increased in cancer: Carbonyl compounds		DI-MS	Case–control	(Li et al. 2015c)
Ovarian carcinoma	Plasma Cancer *n* = *50* Benign control *n* = *50*	Decreased in cancer patients compared to benign patients: alanine, triacylglycerol, phospholipids	Amino acid metabolism Lipid metabolism Phospholipid metabolism	LC–MS	Case–control	(Buas et al. 2016)
Prostate cancer	Serum Cancer *n* = *64* Healthy control *n* = *50*	Panel of 40 metabolites—including fatty acids, amino acids, lysophospholipids and bile acids—can discriminate between healthy and prostate cancer patients.	Amino acid metabolism Lipid metabolism Phospholipid metabolism	LC–MS/MS	Case–control	(Zang et al. 2014)
*(B) Prognostic metabolic markers associated with future cancer incidence:*						
Breast cancer; prostate cancer; colorectal cancer	Plasma Pre-diagnostic samples from cancer patients: Breast *n* = 362 Prostate *n* = 310 Colorectal *n* = *163*	Positive association with cancer: Phosphatidylcholine(30:0) Negative association with cancer: Lysophosphatidylcholines—particularly LPC(18:0)	Phospholipid metabolism	LC–MS/MS	Prognostic case–control	(Kuhn et al. 2016)
Hepatocellular carcinoma	Serum Pre-diagnostic samples from cancer patients *n* = *114* Healthy control cohort *n* = *222*	Positive association with cancer: glutamate, tyrosine, phenylalanine, glucose Negative association with cancer: choline, leucine, isoleucine, glutamine, unsaturated lipids	Amino acid metabolism Energy metabolism Lipid metabolism Phospholipid metabolism	NMR	Prognostic case–control	(Fages et al. 2015)
Pancreatic adenocarcinoma	Plasma Pre-diagnostic samples from cancer patients *n* = *454* Healthy control cohort *n* = *908*	Positive association with cancer: branched chain amino acids—leucine, isoleucine, valine	Amino acid metabolism	LC–MS	Prognostic case–control	(Mayers et al. 2014)
Prostate cancer	Serum Pre-diagnostic samples from cancer patients *n* = *200* Healthy control cohort *n* = *200*	Negative association with cancer: inositol-1-phosphate, citrate, α-ketoglutarate, free fatty acids, phospholipids	Energy metabolism Lipid metabolism Phospholipid metabolism	LC–MS/GC–MS	Prognostic case–control	(Mondul et al. 2015)
*(C) Metabolic markers associated with cancer progression, relapse or remission:*						
Bladder cancer	Urine pre-drug treatment samples from cancer patients *n* = *48* (*n* = *27* continued to stable disease; *n* = *21* progressed to recurrent disease).	Increased in relapse: histidine, tyrosine, tryptophan	Amino acid metabolism	LC–MS/CE-MS	Case–control	(Alberice et al. 2013)
Colorectal cancer	Serum cancer patients *n* = *20*	Increased in disease progression: succinate, N2, N2-dimethylguanosine, adenine, citraconic acid, 1-methylguanosine	Energy metabolism Nucleotide metabolism	LC–MS/MS	Longitudinal	(Zhu et al. 2015)
Myeloma	Serum myeloma patients n = 32 (*n* = *13* entered remission; *n* = *19* relapsed)	Decreased in remission and Increased in relapse: acetylcarnitine, carnitine	Fatty acid β-oxidation	NMR	Longitudinal	(Lodi et al. 2013)
Myeloma	Serum myeloma patients *n* = *27* (*n* = *23* entered remission) Healthy control cohort *n* = *31*	Increased in remission: lysine, citrate, lactate Decreased in remission: glucose	Amino acid metabolism Energy metabolism	NMR	Longitudinal and case–control	(Puchades-Carrasco et al. 2013)

**Fig. 2:**
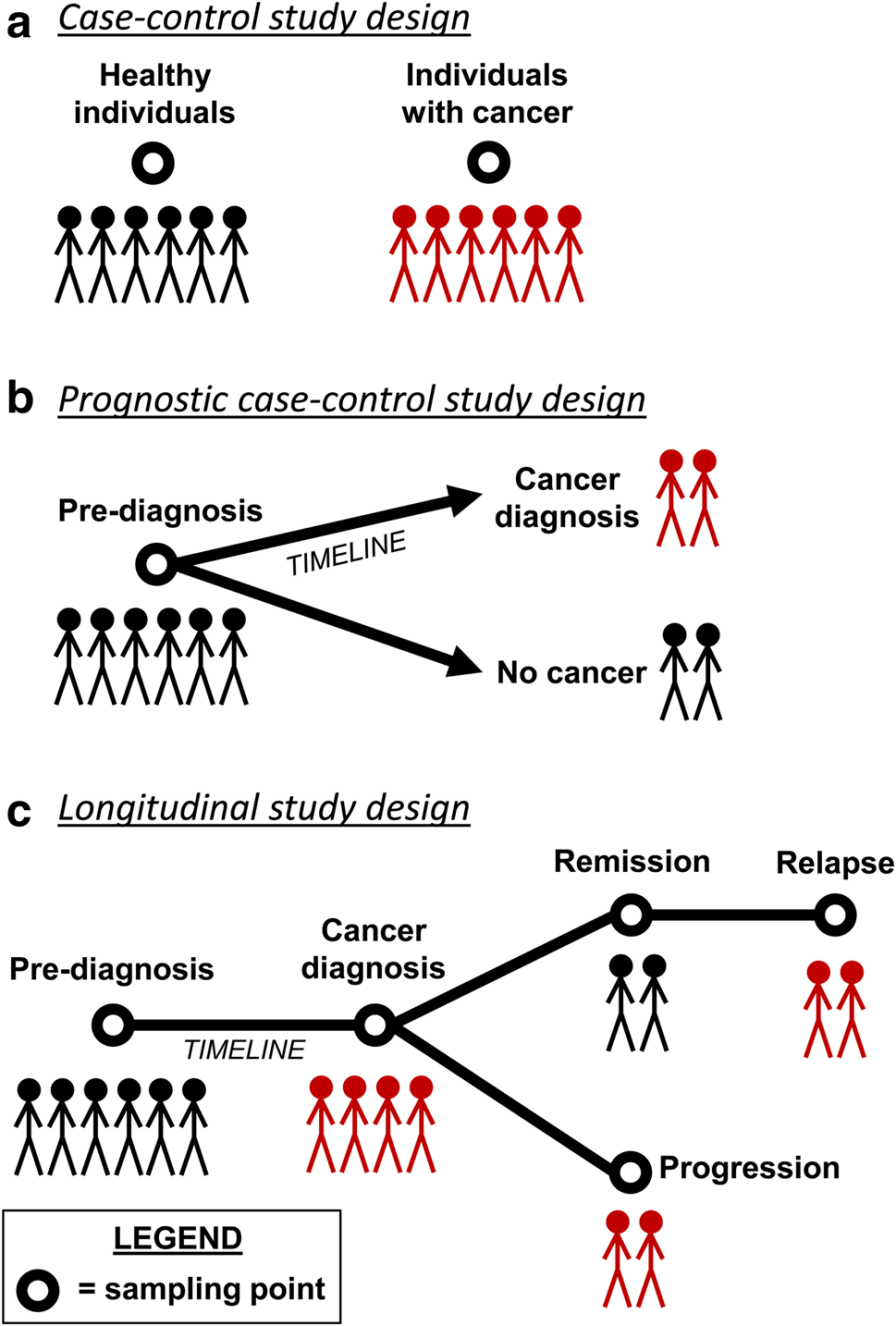
Metabolomics and lipidomics study designs. **a** Case–control studies utilise genetically different cohorts for control subjects and subjects with cancer. **b** Prognostic case–control studies use samples taken from patients before an event, e.g. cancer diagnosis. This enables metabolic features to be correlated with future cancer risk. **c** Longitudinal approaches analyse samples taken from each patient at multiple time-points

Studies are separated according to output—(A) metabolic markers of cancer, (B) prognostic metabolic markers associated with future cancer risk and (C) metabolic markers associated with cancer progression, relapse or remission. For study design description see Fig. 2

Effective study design is important (Fig. 2). Case–control studies compare a cohort of cancer patients against a cohort of healthy patients to identify metabolic markers of disease (Xie et al. 2015). Prognostic case–control studies analyse patient samples taken before cancer diagnosis to identify metabolic signatures that are indicative of cancer risk (Mayers et al. 2014). Longitudinal studies take several samples from patients over a time period—e.g. prior to diagnosis, at diagnosis and in remission—meaning each patient has a control sample to which other sample time-points are compared. Longitudinal studies can be used to identify metabolic markers indicative of (i) cancer prognosis and risk of disease (Cook et al. 2016), (ii) patient remission or relapse (Lodi et al. 2013) and (iii) the mechanism-of-action and success of anticancer drug therapies (Jobard et al. 2015).

### Case–control studies to identify metabolic markers of cancer

Case–control study design is the most commonly used approach to identify metabolic markers associated with cancer (Armitage and Barbas 2014) (Fig. 2). Many of these biomarkers are related to lipid metabolism (Table 1). Free fatty acids and lysophosphatidylcholines were shown to be elevated in serum from lung (Li et al. 2014) and colorectal cancer (Li et al. 2013) patients compared to controls, whilst phospholipid composition was altered in the serum of patients with breast (Yang et al. 2015), colorectal (Li et al. 2013) and ovarian (Buas et al. 2016) cancers. These changes are consistent with the lipogenic phenotype associated with cancer (Menendez and Lupu 2007) and cancer-induced changes to phospholipid composition (Marien et al. 2015). Other lipid changes include increased serum acetylcarnitine and acylcarnitine levels in aggressive chronic lymphocytic leukaemia (CLL) patients relative to indolent CLL patients (Piszcz et al. 2016), and increased carnitine and select acylcarnitines in urine from bladder cancer patients (Jin et al. 2014). This suggests an alteration of mitochondrial fatty acid β-oxidation, which has been shown as an anticancer target (Samudio et al. 2010). Non-lipid metabolic markers of cancer mainly included glycolytic or TCA cycle metabolites (An et al. 2015; Jin et al. 2014; Wittmann et al. 2014). Alanine was identified as a marker in three studies: elevated in gastric cancer (Chan et al. 2016) and leptomeningeal carcinomatosis (An et al. 2015), and decreased in ovarian cancer (Buas et al. 2016). Changeable alanine levels may be related to the glycolytic cancer phenotype where pyruvate can be used to produce alanine and other non-essential amino acids (Munoz-Pinedo et al. 2012).

### Prognostic case–control and longitudinal approaches to identify metabolic markers associated with the risk of developing cancer

With the correct study design, metabolomics and lipidomics can identify metabolic markers that are indicative of future cancer risk. This could allow patients to be treated earlier or enable the design of interventions that delay or prevent cancer onset. For this approach, biological samples are taken from multiple patients without cancer—representing pre-disease baseline metabolism. Patients are then monitored over months/years for incidence of cancer. A prognostic case–control study compares metabolic baseline profiles from individuals who develop cancer against matched control patients who do not develop cancer (Fig. 2; Table 1) (Kuhn et al. 2016; Mayers et al. 2014). A prognostic longitudinal approach requires further sampling from each subject (e.g. on diagnosis, in remission; Fig. 2), which can then be compared to baseline metabolism (Cook et al. 2016). The collection of baseline samples before cancer diagnosis requires years of forward planning or access to archived patient samples. Also, an initial large patient cohort is required to ensure sufficient patients will develop cancer—often several thousand subjects (Kuhn et al. 2016; Mayers et al. 2014). As a result, prognostic studies are less common to standard case–control studies. However, this approach has shown that metabolic markers can indicate cancer risk years before diagnosis (Kuhn et al. 2016), demonstrating it to be clinically very powerful.

Recent prognostic case–control metabolomics studies indicate that blood lipid composition is indicative of future cancer risk (Table 1). Serum levels of lysophosphatidylcholines, particularly LPC(18:0), are negatively associated with breast, prostate and colorectal cancer risk, whereas the phosphatidylcholine(30:0) level was positively associated (Kuhn et al. 2016). A separate study showed that many serum lipids—including free fatty acids and various phospholipids—have a negative association with aggressive prostate cancer (Mondul et al. 2015). A further study showed that levels of unsaturated lipids in the serum were negatively associated with hepatocellular carcinoma incidence (Fages et al. 2015). This demonstrates the importance of lipid metabolism in cancer and is consistent with findings from case–control studies (above). Considering non-lipid prognostic markers (Table 1), altered serum levels of energy-related metabolites were associated with cancer: α-ketoglutarate and citrate were negatively associated with prostate cancer (Mondul et al. 2015) and glucose positively associated with hepatocellular carcinoma (Fages et al. 2015). Elevated serum levels of branched-chain amino acids are associated with a >twofold increased risk of pancreatic ductal adenocarcinoma (Mayers et al. 2014), which is in contrast to hepatocellular carcinoma where the opposite was reported (Fages et al. 2015). The inconsistency may be explained by the effect the cancer has on the function of the disease tissue—in pancreatic ductal adenocarcinoma, altered pancreas function changes glucose metabolism leading to whole-body protein breakdown and elevated branched chain amino acids (Mayers et al. 2014). This emphasises the need to consider all bodily processes when interpreting data acquired from patient biofluids.

Currently, the application of longitudinal metabolomics to monitor cancer prognosis is rare. However, this approach has been used to analyse mouse urine and can successfully predict the incidence of several different types of cancer (Cook et al. 2016). A notable advantage of a longitudinal approach over a prognostic case–control approach is the analysis of metabolism both before diagnosis and on diagnosis. This establishes metabolic indicators of cancer risk while also understanding how and why these metabolic processes change on cancer initiation.

### Identification of metabolic markers of cancer progression, relapse and remission

Longitudinal metabolomics is ideal to monitor cancer progression, relapse and remission. Here, patient samples are collected at cancer diagnosis and on several occasions afterwards (Fig. 2). Analysis of the samples aims to identify metabolic features that correlate with—and can therefore be indicative of—relapse or remission (Table 1). Longitudinal studies have shown TCA cycle intermediates and RNA degradation products to decrease in colorectal cancer patients’ serum once remission or stable disease has been reached (Zhu et al. 2015). Serum levels of carnitine and acetylcarnitine were lower in remission and increased in relapse in multiple myeloma patients (Lodi et al. 2013). This indicates that mitochondrial β-oxidation is altered at different cancer stages, which is consistent with case–control studies above (Jin et al. 2014; Piszcz et al. 2016). Case–control metabolomics has also been applied to greater understand cancer progression including a study where aromatic amino acid levels in patient urine samples were shown to be indicative of bladder cancer disease stage (Alberice et al. 2013).

A key consideration when investigating cancer progression is the distinction between drug-induced metabolic changes—as cancer patients will likely receive therapy on diagnosis—and cancer-induced metabolic changes. This issue is highlighted in a multiple myeloma study where the biomarkers that were able to distinguish patients in remission from those at diagnosis (glucose, citrate and lactate) were likely attributable to bortezomib drug therapy (Puchades-Carrasco et al. 2013). The value of these markers as indicators of disease remission is unclear without an understanding of the therapeutic drug metabolism.

## Using metabolomics and lipidomics to understand the mechanism-of-action of anticancer therapeutics

Metabolomics and lipidomics can be used to elucidate the metabolic mechanism-of-action of anticancer therapeutics. This information has the potential to improve therapies and understand why some patients respond but others do not (Nicholson et al. 2011; Holmes et al. 2015). The following sections highlight how metabolomics and lipidomics contribute to understanding drug action—including optimising drug delivery strategies, understanding drug resistance and exploration of nutraceuticals for anticancer therapy.

### Anticancer therapeutics

Metabolomic and lipidomic investigation of anticancer therapeutics has been applied to patient samples and in vitro models (He et al. 2015; Schuler et al. 2015). The most commonly studied drug is metformin, which was originally intended to treat type II diabetes but also has anticancer activity arising from its inhibition of mitochondrial complex I and production of energetic stress (Pernicova and Korbonits 2014). Metabolomics analysis of serum samples from metformin-treated breast cancer patients revealed disruptions to glucose and insulin metabolism (Lord et al. 2015). Further metabolomics analyses indicated that metformin also alters methionine and folate cycles to decrease nucleotide synthesis, which may further contribute to the anticancer activity (Jara and López-Muñoz 2015). Additional examples of metabolomic and lipidomic investigation of anticancer therapeutics are detailed in Table 2.

**Table 2:** Examples where metabolomics or lipidomics has been used to understand the mechanism-of-action of anticancer therapies

Treatment	Experimental design and study size	Metabolic response	Outcome	Reference
Metformin	Goal: preoperative study of endometrial cancer patients to evaluate metformin action Samples (*n* = *20*): Serum pre- and post-treatment tumour samples post-treatment Approach: LC–MS global profiling	Patients who responded to metformin showed increased lipolysis, fatty acid oxidation and glycogen metabolism	Metformin could be a viable treatment for obese individuals with endometrial cancer	(Schuler et al. 2015)
Metformin	Goal: to study the metabolic effect of metformin treatment Samples (*n* = *5*): Human-derived LoVo cells Approach: Metabolic profiling by GC–MS & LC–MS, transcriptomics	Considerable metabolic changes in carbohydrate, lipid, amino acid, vitamin and nucleotide metabolism after metformin treatment. 100-1000 s differentially expressed genes involving cancer signalling and cell energy metabolism mechanisms	Metformin supresses proliferation of LoVo cells, likely through modulation of cell energy metabolism at both transcriptomic and metabolomics levels	(He et al. 2015)
Asprin	Goal: Highlight potential as anti-cancer treatment Samples (*n* = *40*): Plasma Approach: Profiling by GC–MS & LC–MS	Aspirin decreases levels of the onco-metabolite, 2-hydroxyglutarate	Aspirin appears to have anti-cancer properties and thus may be an effective treatment for some cancers	(Liesenfeld et al. 2016)
Daunorubicin	Goal: Study of resistance by comparison to sensitive cells Samples (*n* = *5*): P-glycoprotein overexpressing T cell acute lymphoblastic leukaemia cells Approach: Untargeted metabolomics analysis utilising LC–MS and validation of selective targets	Drug-resistant cells were metabolically different to drug-sensitive cells. Resistant cells have the following traits: reduced dependence on glutamine higher demand for glucose altered rate of fatty acid β-oxidation decreased capacity for pantothenic acid uptake	Targeting the metabolic changes observed in drug-resistant cells has the potential to increase anticancer drug efficacy	(Stäubert et al. 2015)
Doxatel	Goal: Compare the metabolism of drug-resistant tissue to drug-sensitive tissue Samples (*n* = *5*): BRCA1-mutated mouse mammary tumours Approach: Magnetic resonance spectroscopy	Choline-containing metabolites are higher in concentration in resistant tissue compared to sensitive tissue. After treatment, the concentration of choline metabolites increases in drug-sensitive tissue.	Pre- and post-treatment tissue levels of choline compounds have potential to predict response to treatment	(van Asten et al. 2015)
Platinum	Goal: Compare the metabolic profile of drug-resistant cells to drug-sensitive cells Samples (*n* = *5*): Ovarian cancer cells Approach: Profiling by GC–MS & LC–MS	In platinum-resistant cells, 70 metabolites were increased and 109 metabolites decreased. The metabolic pathway with the most alterations was cysteine & methionine metabolism	Resistance to platinum is linked to cysteine and methionine metabolism. This may be related to glutathione synthesis and how cells cope with oxidative stress.	(Poisson et al. 2015)
Bezafibrate and medroxyprogesterone acetate combination	Goal: Understand the anticancer mechanism of action of the drugs Samples (lipidomics *n* = *7*): Acute myeloid leukaemia and Burkitt’s lymphoma cell lines Approach: Profiling by DIMS & ^13^C-isotope pulse-chase isotope labelling DIMS	Phospholipids with polyunsaturated acyl chains increase after treatment, while those with saturated or monounsaturated acyl chains decrease. Fatty acid biosynthesis from glucose—in particular that of monounsaturated fatty acids—was decreased	Drug-induced decrease in monounsaturated fatty acid synthesis plays a role in the anticancer activity of this redeployed drug combination	(Southam et al. 2015)

### Drug redeployment

Drug redeployment (also known as drug repositioning or drug repurposing) involves the use of existing drug(s) in a situation it was not originally intended (Ashburn and Thor 2004). Candidate drugs would typically be identified by screening a panel of licenced drugs for anticancer effect. The benefit of this approach is that drug pharmacokinetics and toxicity are already known, eliminating the need for early stage clinical trials. However, the exact anticancer mechanism of the drug is often unknown. Metabolomics and lipidomics has been used to understand the metabolic mechanism-of-action of redeployed anticancer drugs, e.g. metformin (see above), aspirin (Liesenfeld et al. 2016) and bezafibrate/medroxyprogesterone acetate (Southam et al. 2015) (Table 2). There are, however, important considerations when using metabolomics and lipidomics to elucidate drug mechanism. Firstly, it can be challenging to distinguish anticancer metabolic effects of the drug from the whole body metabolic response to the drug. In this case, further experimentation is required to prove that the metabolic changes actually correlate to anticancer effect. Additionally, redeployed drugs are often used as combinations and different doses compared to their original intended prescribed dose (Khanim et al. 2009). This could alter drug effects and/or increase the number of metabolic processes that are perturbed, making it more difficult to distinguish anticancer metabolic effects from the general metabolic perturbations caused by the drugs.

### Stratified and personalised medicine

Stratified medicine aims to predict whether cancer patients will respond to therapy (Trusheim et al. 2007). Using a prognostic study design (Fig. 2b—where the outcome is drug response rather than cancer incidence) it is possible to identify metabolic profiles predictive of drug response, which could be used to personalise treatments for individual patients (Nicholson et al. 2011). Adopting this approach, metabolomics has been utilised to understand how mitomycin C should be used in the treatment of pancreatic cancer (Navarrete et al. 2014). In this study, patient pancreatic adenocarcinoma cells were xenografted on to a murine tumour model and then cells were treated with mitomycin C, rapamycin or a combination of both. Mitomycin C had a greater anticancer effect than rapamycin alone or the combined drugs. The authors propose that the effectiveness of mitomycin C alone was due to its effect on central carbon metabolism. Metabolomics has also been used to stratify metformin treatment. It was shown that cells with mutated isocitrate dehydrogenase 1 (IDH1) have a metabolic phenotype that increases their vulnerability to metformin (Cuyàs et al. 2015). This suggests that metformin would be most effective against tumours with IDH1 mutations [e.g. brain tumours and acute myeloid leukaemia (Balss et al. 2008; Schnittger et al. 2010].

### Novel drug administration strategies

Advancements in drug administration can allow drugs to reach the target cancer tissue more effectively, e.g. polymer-nanoparticle-encapsulation can co-deliver two drugs—doxorubicin and paclitaxel—to cancer cells to maximise the synergistic effect of the drugs (Wang et al. 2011). NMR metabolomics has been used to investigate systemic toxic effect(s) of the polymer-nanoparticle-encapsulation material used to deliver doxorubicin and paclitaxel compared to the free forms of the drugs in mice (Song et al. 2015). The encapsulation material induced a slight and temporal metabolic effect in the mice—supporting this as a low toxicity approach—while encapsulation decreased the toxicity of the drugs on the heart compared to administration of free drugs (Song et al. 2015).

### Drug resistance

Cancer cells often develop resistance towards drug therapies (Gottesman 2002). Understanding why resistance occurs could allow the therapy to be modified to overcome the resistance. Metabolomics has informed on the resistance mechanism of some anticancer drugs. Resistance to the chemotherapeutic agent temozolomide is common during the treatment of glioblastoma multiforme (St-Coeur et al. 2015). Metabolomics has been used to understand the mechanism of resistance in glioblastoma multiforme cell lines and primary tumours, and also to explore the metabolic effects of the temozolomide-sensitizing agent, Lomeguatrib (St-Coeur et al. 2015). Glucose, citrate and isocitrate were increased in resistant cells, whereas alanine, choline, creatine and phosphorylcholine were increased in sensitive cells, demonstrating a metabolic aspect to the drug resistance (St-Coeur et al. 2015). These metabolic signatures could predict drug responses and, once the metabolic perturbations are understood, could help contribute to the improvement of therapies in glioblastoma multiforme. Additionally, the imaging approach, time-of-flight secondary ion mass spectrometry (ToF–SIMS), has been used to study metabolic regulation of hypoxia-induced chemoresistance to doxorubicin treatment of multicellular tumour spheroids (Kotze et al. 2013). Cholesterol and diacylglycerols were implicated as response markers of treatment in the hypoxic regions, which suggested that lipids play a role in drug response and resistance in hypoxic regions of tumours (Kotze et al. 2013).

### Nutraceutical cancer treatments

Natural plant extracts or plant-derived nutrients can have anticancer properties (Babbar et al. 2015), and therefore often offer a viable alternative to pharmaceuticals. Metabolomics and lipidomics can aid with the elucidation of the mechanism-of-action of such compounds. Volatile oil extracted from *Saussurea lappa* Decne in addition to costunolide and dehydrocostus lactone isolated from the oil have shown anticancer properties against breast cancer cells (Peng et al. 2015). Metabolomics of serum and urine samples from MCF-7 xenograft mice revealed that the oil and the extracted compounds can reverse the metabolic phenotype associated with the MCF-7 xenograft (initial MCF-7 xenograft increases glycolysis and steroid hormone metabolism, and decreases unsaturated fatty acid metabolism) (Peng et al. 2015). Halofuginone, extracted from *Dichroa febrifuga*, can inhibit colorectal cancer growth in vitro and in vivo (Chen et al. 2015). Metabolic flux analysis showed halofuginone to decrease glycolytic and TCA cycle intermediates, which was correlated with reduced GLUT 1 activity and glucose uptake (Chen et al. 2015). Lipidomics revealed a decrease in phospholipids, ceramide and sphingomyelin after treatment, which was consistent with the reported halofuginone-induced decrease of fatty acid synthase expression (Chen et al. 2015). These findings suggest that halofuginone can target the known metabolic cancer targets aerobic glycolysis and fatty acid biosynthesis. Flexibilide isolated from coral (*Sinularia flexibilis*) has anticancer properties (Gao et al. 2016). Metabolomics analysis of flexibilide-treated HCT-116 colorectal cancer cells indicated that the compound modulates sphingolipid metabolism, amino acid metabolism, phospholipid metabolism and pyrimidine metabolism, which the authors suggest may be associated with the anti-tumour activity (Gao et al. 2016). Nutmeg has also been studied for its effect against colorectal carcinoma (Li et al. 2015a). Serum metabolomics revealed that colon cancer bearing mice have elevated levels of uremic toxins cresol sulfate, cresol glucuronide, indoxyl sulfate and phenyl sulfate, which are likely generated from gut microbiota and are implicated in tumorigenesis (Li et al. 2015a). Nutmeg has been shown to attenuate the serum levels of these compounds, potentially reflecting the antibacterial and anticancer properties of nutmeg (Li et al. 2015a). This study highlights that it is important to understand the role of gut microbiota in cancer—an expanding and important research topic. It has been shown that human colorectal cancer cells carrying KRAS and BRAF mutations—giving them a highly glycolytic phenotype—can be selectively killed by high doses of vitamin C (Yun et al. 2015). Metabolomics revealed that vitamin C causes pentose phosphate pathway metabolites and glycolytic intermediates located up-stream of glyceraldehyde 3-phosphate dehydrogenase (GAPDH) to increase in KRAS and BRAF mutated colorectal cancer cells, whereas metabolites down-stream of GAPDH were decreased (Yun et al. 2015). Vitamin C was subsequently demonstrated to inhibit the GAPDH enzyme through the accumulation of reactive oxygen species (Yun et al. 2015).

## Conclusions and future perspectives

Metabolomics and lipidomics are important tools for cancer research. They can be used to discover biomarkers indicative of patient prognosis, diagnosis and treatment efficacy, and to aid in the elucidation of the mechanism-of-action of novel and existing anticancer therapeutics. To identify robust and clinically useful biomarkers effective study design is essential. Prognostic studies—where samples are taken prior to cancer diagnosis—can identify metabolic markers indicative of future cancer risk. Longitudinal studies—involving analysis of multiple samples taken the each patient over a time period—is a good strategy to investigate the metabolic aspects of cancer progression. Considering anticancer therapy development, metabolomics and lipidomics have contributed to the development and understanding of pharmaceutical therapies, nutraceutical therapies and novel drug delivery strategies. Key future research applications for metabolomics and lipidomics are to investigate the role of gut microbiota in cancer and to better understand how metabolic therapies can be tailored using a stratified medicine approach. Understanding gut microbiota in cancer is particularly important given that this can alter the metabolic response to drug therapies (Li et al. 2015b) and also the efficacy anticancer treatment (Vétizou et al. 2015).

## References

[CR1] Ahn CS, Metallo CM (2015). Mitochondria as biosynthetic factories for cancer proliferation. Cancer Metabolism.

[CR2] Alberice JV, Amaral AFS, Armitage EG, Lorente JA, Algaba F, Carrilho E, Marquez M, Garcia A, Malats N, Barbas C (2013). Searching for urine biomarkers of bladder cancer recurrence using a liquid chromatography-mass spectrometry and capillary electrophoresis-mass spectrometry metabolomics approach. Journal of Chromatography A.

[CR3] An YJ, Cho HR, Kim TM, Keam B, Kim JW, Wen H, Park CK, Lee SH, Im SA, Kim JE, Choi SH, Park S (2015). An NMR metabolomics approach for the diagnosis of leptomeningeal carcinomatosis in lung adenocarcinoma cancer patients. International Journal of Cancer.

[CR4] Armitage EG, Barbas C (2014). Metabolomics in cancer biomarker discovery: current trends and future perspectives. Journal of Pharmaceutical and Biomedical Analysis.

[CR5] Ashburn TT, Thor KB (2004). Drug repositioning: identifying and developing new uses for existing drugs. Nature Reviews Drug Discovery.

[CR6] Babbar N, Oberoi HS, Sandhu SK (2015). Therapeutic and nutraceutical potential of bioactive compounds extracted from fruit residues. Critical Reviews in Food Science and Nutrition.

[CR7] Balog J, Sasi-Szabo L, Kinross J, Lewis MR, Muirhead LJ, Veselkov K, Mirnezami R, Dezso B, Damjanovich L, Darzi A, Nicholson JK, Takats Z (2013). Intraoperative tissue identification using rapid evaporative ionization mass spectrometry. Science Translational Medicine.

[CR8] Balss J, Meyer J, Mueller W, Korshunov A, Hartmann C, von Deimling A (2008). Analysis of the IDH1 codon 132 mutation in brain tumors. Acta Neuropathologica.

[CR9] Bertini I, Cacciatore S, Jensen BV, Schou JV, Johansen JS, Kruhoffer M, Luchinat C, Nielsen DL, Turano P (2012). Metabolomic NMR fingerprinting to identify and predict survival of patients with metastatic colorectal cancer. Cancer Research.

[CR10] Boroughs LK, Deberardinis RJ (2015). Metabolic pathways promoting cancer cell survival and growth. Nature Cell Biology.

[CR11] Buas MF, Gu H, Djukovic D, Zhu J, Drescher CW, Urban N, Raftery D, Li CI (2016). Identification of novel candidate plasma metabolite biomarkers for distinguishing serous ovarian carcinoma and benign serous ovarian tumors. Gynecologic Oncology.

[CR12] Chan AW, Mercier P, Schiller D, Bailey R, Robbins S, Eurich DT, Sawyer MB, Broadhurst D (2016). (1)H-NMR urinary metabolomic profiling for diagnosis of gastric cancer. British Journal of Cancer.

[CR13] Chen G-Q, Tang C-F, Shi X-K, Lin C-Y, Fatima S, Pan X-H, Yang D-J, Zhang G, Lu A-P, Lin S-H, Bian Z-X (2015). Halofuginone inhibits colorectal cancer growth through suppression of Akt/mTORC1 signaling and glucose metabolism. Oncotarget.

[CR14] Cook JA, Chandramouli GVR, Anver MR, Sowers AL, Thetford A, Krausz KW, Gonzalez FJ, Mitchell JB, Patterson AD (2016). Mass spectrometry-based metabolomics identifies longitudinal urinary metabolite profiles predictive of radiation-induced cancer. Cancer Research.

[CR15] Currie E, Schulze A, Zechner R, Walther TC, Farese Jr RV (2013). Cellular fatty acid metabolism and cancer. Cell Metabolism.

[CR16] Cuyàs E, Fernández-Arroyo S, Corominas-Faja B, Rodríguez-Gallego E, Bosch-Barrera J, Martin-Castillo B, de Llorens R, Joven J, Menendez JA (2015). Oncometabolic mutation IDH1 R132H confers a metformin-hypersensitive phenotype. Oncotarget.

[CR17] Deberardinis RJ, Mancuso A, Daikhin E, Nissim I, Yudkoff M, Wehrli S, Thompson CB (2007). Beyond aerobic glycolysis: transformed cells can engage in glutamine metabolism that exceeds the requirement for protein and nucleotide synthesis. Proceedings of the National Academy of Sciences of the United States of America.

[CR18] Doherty JR, Cleveland JL (2013). Targeting lactate metabolism for cancer therapeutics. Journal of Clinical Investigation.

[CR19] Fages A, Duarte-Salles T, Stepien M, Ferrari P, Fedirko V, Pontoizeau C, Trichopoulou A, Aleksandrova K, Tjønneland A, Olsen A, Clavel-Chapelon F (2015). Metabolomic profiles of hepatocellular carcinoma in a European prospective cohort. BMC Medicine.

[CR20] Gao D, Wang Y, Xie W, Yang T, Jiang Y, Guo Y, Guan J, Liu H (2016). Metabolomics study on the antitumor effect of marine natural compound flexibilide in HCT-116 colon cancer cell line. Journal of Chromatography B-Analytical Technologies in the Biomedical and Life Sciences.

[CR21] Gill SK, Wilson M, Davies NP, Macpherson L, English M, Arvanitis TN, Peet AC (2014). Diagnosing relapse in childrens brain tumors using metabolite profiles. Neuro-Oncology.

[CR22] Gottesman MM (2002). Mechanisms of cancer drug resistance. Annual Review of Medicine.

[CR23] Griffiths RL, Dexter A, Creese AJ, Cooper HJ (2015). Liquid extraction surface analysis field asymmetric waveform ion mobility spectrometry mass spectrometry for the analysis of dried blood spots. Analyst.

[CR24] He J, Wang K, Zheng N, Qiu Y, Xie G, Su M, Jia W, Liiiii H (2015). Metformin suppressed the proliferation of LoVo cells and induced a time-dependent metabolic and transcriptional alteration. Sci Rep..

[CR25] Hensley CT, Faubert B, Yuan Q, Lev-Cohain N, Jin E, Kim J, Jiang L, Ko B, Skelton R, Loudat L, Wodzak M, Klimko C, McMillan E, Butt Y, Ni M, Oliver D, Torrealba J, Malloy CR, Kernstine K, Lenkinski RE, Deberardinis RJ (2016). Metabolic heterogeneity in human lung tumors. Cell.

[CR26] Holmes E, Wijeyesekera A, Taylor-Robinson SD, Nicholson JK (2015). The promise of metabolic phenotyping in gastroenterology and hepatology. Nat Rev Gastroenterol Hepatol.

[CR27] Jara J, López-Muñoz R (2015). Metformin and cancer: between the bioenergetic disturbances and the antifolate activity. Pharmacological Research.

[CR28] Jin X, Yun SJ, Jeong P, Kim IY, Kim WJ, Park S (2014). Diagnosis of bladder cancer and prediction of survival by urinary metabolomics. Oncotarget.

[CR29] Jobard E, Blanc E, Negrier S, Escudier B, Gravis G, Chevreau C, Elena-Herrmann B, Tredan O (2015). A serum metabolomic fingerprint of bevacizumab and temsirolimus combination as first-line treatment of metastatic renal cell carcinoma. British Journal of Cancer.

[CR30] Kaddurah-Daouk R, Kristal BS, Weinshilboum RM (2008). Metabolomics: a global biochemical approach to drug response and disease. Annual Review of Pharmacology and Toxicology.

[CR31] Kamphorst JJ, Chung MK, Fan J, Rabinowitz JD (2014). Quantitative analysis of acetyl-CoA production in hypoxic cancer cells reveals substantial contribution from acetate. Cancer and Metabolism.

[CR32] Kamphorst JJ, Cross JR, Fan J, de Stanchina E, Mathew R, White EP, Thompson CB, Rabinowitz JD (2013). Hypoxic and Ras-transformed cells support growth by scavenging unsaturated fatty acids from lysophospholipids. Proceedings of the National Academy of Sciences of the United States of America.

[CR33] Khanim FL, Hayden RE, Birtwistle J, Lodi A, Tiziani S, Davies NJ, Ride JP, Viant MR, Gunther UL, Mountford JC, Schrewe H, Green RM, Murray JA, Drayson MT, Bunce CM (2009). Combined bezafibrate and medroxyprogesterone acetate: potential novel therapy for acute myeloid leukaemia. PLoS ONE.

[CR34] Kimmelman AC (2015). Metabolic Dependencies in RAS-Driven Cancers. Clinical Cancer Research.

[CR35] King A, Selak MA, Gottlieb E (2006). Succinate dehydrogenase and fumarate hydratase: linking mitochondrial dysfunction and cancer. Oncogene.

[CR36] Koczula KM, Ludwig C, Hayden R, Cronin L, Pratt G, Parry H, Tennant D, Drayson M, Bunce CM, Khanim FL, Guenther UL (2016). Metabolic plasticity in CLL: adaptation to the hypoxic niche. Leukemia.

[CR37] Kotze HL, Armitage EG, Fletcher JS, Henderson A, Williams KJ, Lockyer NP, Vickerman JC (2013). ToF-SIMS as a tool for metabolic profiling small biomolecules in cancer systems. Surface and Interface Analysis.

[CR38] Krasny L, Hoffmann F, Ernst G, Trede D, Alexandrov T, Havlicek V, Guntinas-Lichius O, von Eggeling F, Crecelius AC (2015). Spatial segmentation of MALDI FT-ICR MSI Data: a powerful tool to explore the head and neck tumor in situ lipidome. Journal of the American Society for Mass Spectrometry.

[CR39] Kuhn T, Floegel A, Sookthai D, Johnson T, Rolle-Kampczyk U, Otto W, von Bergen M, Boeing H, Kaaks R (2016). Higher plasma levels of lysophosphatidylcholine 18:0 are related to a lower risk of common cancers in a prospective metabolomics study. BMC Medicine.

[CR40] Li H, He J, Jia W (2016). The influence of gut microbiota on drug metabolism and toxicity. Expert Opinion on Drug Metabolism and Toxicology.

[CR41] Li F, Qin XZ, Chen HQ, Qiu L, Guo YM, Liu H, Chen GQ, Song GG, Wang XD, Li FJ, Guo S, Wang BH, Li ZL (2013). Lipid profiling for early diagnosis and progression of colorectal cancer using direct-infusion electrospray ionization Fourier transform ion cyclotron resonance mass spectrometry. Rapid Communications in Mass Spectrometry.

[CR42] Li YJ, Song X, Zhao XJ, Zou LJ, Xu GW (2014). Serum metabolic profiling study of lung cancer using ultra high performance liquid chromatography/quadrupole time-of-flight mass spectrometry. Journal of Chromatography B-Analytical Technologies in the Biomedical and Life Sciences.

[CR43] Li MX, Yang DK, Brock G, Knipp RJ, Bousamra M, Nantz MH, Fu XA (2015). Breath carbonyl compounds as biomarkers of lung cancer. Lung Cancer.

[CR44] Li F, Yang X-W, Krausz KW, Nichols RG, Xu W, Patterson AD, Gonzalez FJ (2015). Modulation of Colon Cancer by Nutmeg. Journal of Proteome Research.

[CR45] Liesenfeld DB, Botma A, Habermann N, Toth R, Weigel C, Popanda O, Klika KD, Potter JD, Lampe JW, Ulrich CM (2016). Aspirin reduces plasma concentrations of the oncometabolite 2-hydroxyglutarate: results of a randomized, double-blind, crossover trial. Cancer Epidemiology, Biomarkers and Prevention.

[CR46] Lindon JC, Holmes E, Nicholson JK (2006). Metabonomics techniques and applications to pharmaceutical research and development. Pharmaceutical Research.

[CR47] Lodi A, Tiziani S, Khanim FL, Günther UL, Viant MR, Morgan GJ, Bunce CM, Drayson MT (2013). Proton NMR-based metabolite analyses of archived serial paired serum and urine samples from myeloma patients at different stages of disease activity identifies acetylcarnitine as a novel marker of active disease. PLoS ONE.

[CR48] Lohr JG, Stojanov P, Carter SL, Cruz-Gordillo P, Lawrence MS, Auclair D, Sougnez C, Knoechel B, Gould J, Saksena G, Cibulskis K (2014). Widespread genetic heterogeneity in multiple myeloma: implications for targeted therapy. Cancer Cell.

[CR49] Lord S, Patel N, Liu D, Fenwick J, Gleeson F, Buffa F, Harris A (2015). Neoadjuvant window studies of metformin and biomarker development for drugs targeting cancer metabolism. Journal of the National Cancer Institute Monographs.

[CR50] Mabbott S, Correa E, Cowcher DP, Allwood JW, Goodacre R (2013). Optimization of parameters for the quantitative surface-enhanced raman scattering detection of mephedrone using a fractional factorial design and a portable Raman spectrometer. Analytical Chemistry.

[CR51] Marien E, Meister M, Muley T, Fieuws S, Bordel S, Derua R, Spraggins J, van de Plas R, Dehairs J, Wouters J, Bagadi M, Dienemann H, Thomas M, Schnabel PA, Caprioli RM, Waelkens E, Swinnen JV (2015). Non-small cell lung cancer is characterized by dramatic changes in phospholipid profiles. International Journal of Cancer.

[CR52] Mayers JR, Wu C, Clish CB, Kraft P, Torrence ME, Fiske BP, Yuan C, Bao Y, Townsend MK, Tworoger SS, Davidson SM, Papagiannakopoulos T, Yang A, Dayton TL, Ogino S, Stampfer MJ, Giovannucci EL, Qian ZR, Rubinson DA, Ma J, Sesso HD, Gaziano JM, Cochrane BB, Liu SM, Wactawski-Wende J, Manson JE, Pollak MN, Kimmelman AC, Souza A, Pierce K, Wang TJ, Gerszten RE, Fuchs CS, Vander Heiden MG, WOLPIN BM (2014). Elevation of circulating branched-chain amino acids is an early event in human pancreatic adenocarcinoma development. Nature Medicine.

[CR53] Menendez JA, Lupu R (2007). Fatty acid synthase and the lipogenic phenotype in cancer pathogenesis. Nature Reviews Cancer.

[CR54] Migita T, Ruiz S, Fornari A, Fiorentino M, Priolo C, Zadra G, Inazuka F, Grisanzio C, Palescandolo E, Shin E, Fiore C, Xie W, Kung AL, Febbo PG, Subramanian A, Mucci L, Ma J, Signoretti S, Stampfer M, Hahn WC, Finn S, Loda M (2009). Fatty acid synthase: a metabolic enzyme and candidate oncogene in prostate cancer. Journal of the National Cancer Institute.

[CR55] Mondul AM, Moore SC, Weinstein SJ, Karoly ED, Sampson JN, Albanes D (2015). Metabolomic analysis of prostate cancer risk in a prospective cohort: the alpha-tocolpherol, beta-carotene cancer prevention (ATBC) study. International Journal of Cancer.

[CR56] Munoz-Pinedo C, El Mjiyad N, Ricci JE (2012). Cancer metabolism: current perspectives and future directions. Cell Death and Disease.

[CR57] Navarrete A, Armitage EG, Musteanu M, García A, Mastrangelo A, Bujak R, López-Casas PP, Hidalgo M, Barbas C (2014). Metabolomic evaluation of Mitomycin C and rapamycin in a personalized treatment of pancreatic cancer. Pharmacology Research and Perspectives..

[CR58] Nicholson JK, Wilson ID, Lindon JC (2011). Pharmacometabonomics as an effector for personalized medicine. Pharmacogenomics.

[CR59] Patra KC, Hay N (2014). The pentose phosphate pathway and cancer. Trends in Biochemical Sciences.

[CR60] Peng Z-X, Wang Y, Gu X, Xue Y, Wu Q, Zhou J-Y, Yan C (2015). Metabolic transformation of breast cancer in a MCF-7 xenograft mouse model and inhibitory effect of volatile oil from Saussurea lappa Decne treatment. Metabolomics.

[CR61] Pernicova I, Korbonits M (2014). Metformin-mode of action and clinical implications for diabetes and cancer. Nature Reviews Endocrinology.

[CR62] Piszcz J, Armitage EG, Ferrarini A, Rupérez FJ, Kulczynska A, Bolkun L, Kloczko J, Kretowski A, Urbanowicz A, Ciborowski M, Barbas C (2016). To treat or not to treat: metabolomics reveals biomarkers for treatment indication in chronic lymphocytic leukaemia patients. Oncotarget..

[CR63] Poisson LM, Munkarah A, Madi H, Datta I, Hensley-Alford S, Tebbe C, Buekers T, Giri S, Rattan R (2015). A metabolomic approach to identifying platinum resistance in ovarian cancer. Journal of Ovarian Research.

[CR64] Puchades-Carrasco L, Lecumberri R, Martinez-Lopez J, Lahuerta JJ, Mateos MV, Prosper F, San-Miguel JF, Pineda-Lucena A (2013). Multiple myeloma patients have a specific serum metabolomic profile that changes after achieving complete remission. Clinical Cancer Research.

[CR65] Ren SC, Shao YP, Zhao XJ, Hong CS, Wang FB, Lu X, Li J, Ye GZ, Yan M, Zhuang ZP, Xu CL, Xu GW, Sun YH (2016). Integration of metabolomics and transcriptomics reveals major metabolic pathways and potential biomarker involved in prostate cancer. Molecular and Cellular Proteomics.

[CR66] Rocha CM, Barros AS, Goodfellow BJ, Carreira IM, Gomes A, Sousa V, Bernardo J, Carvalho L, Gil AM, Duarte IF (2015). NMR metabolomics of human lung tumours reveals distinct metabolic signatures for adenocarcinoma and squamous cell carcinoma. Carcinogenesis.

[CR67] Rodrigues TB, Serrao EM, Kennedy BW, Hu DE, Kettunen MI, Brindle KM (2014). Magnetic resonance imaging of tumor glycolysis using hyperpolarized 13C-labeled glucose. Nature Medicine.

[CR68] Saito K, Matsumoto S, Takakusagi Y, Matsuo M, Morris HD, Lizak MJ, Munasinghe JP, Devasahayam N, Subramanian S, Mitchell JB, Krishna MC (2015). C-13-MR spectroscopic imaging with hyperpolarized 1-C-13 pyruvate detects early response to radiotherapy in SCC tumors and HT-29 tumors. Clinical Cancer Research.

[CR69] Samudio I, Harmancey R, Fiegl M, Kantarjian H, Konopleva M, Korchin B, Kaluarachchi K, Bornmann W, Duvvuri S, Taegtmeyer H, Andreeff M (2010). Pharmacologic inhibition of fatty acid oxidation sensitizes human leukemia cells to apoptosis induction. Journal of Clinical Investigation.

[CR70] Sancho P, Burgos-Ramos E, Tavera A, Kheir TB, Jagust P, Schoenhals M, Barneda D, Sellers K, Campos-Olivas R, Graña O, Viera CR (2015). MYC/PGC-1α balance determines the metabolic phenotype and plasticity of pancreatic cancer stem cells. Cell Metabolism.

[CR71] Schaefer K-C, Denes J, Albrecht K, Szaniszlo T, Balog J, Skoumal R, Katona M, Toth M, Balogh L, Takats Z (2009). In vivo, in situ tissue analysis using rapid evaporative ionization mass spectrometry. Angewandte Chemie-International Edition.

[CR72] Schnittger S, Haferlach C, Ulke M, Alpermann T, Kern W, Haferlach T (2010). IDH1 mutations are detected in 6.6% of 1414 AML patients and are associated with intermediate risk karyotype and unfavorable prognosis in adults younger than 60 years and unmutated NPM1 status. Blood.

[CR73] Schug ZT, Peck B, Jones DT, Zhang QF, Grosskurth S, Alam IS, Goodwin LM, Smethurst E, Mason S, Blyth K, McGarry L, James D, Shanks E, Kalna G, Saunders RE, Jiang M, Howell M, Lassailly F, Thin MZ, Spencer-Dene B, Stamp G, van den Broek NJF, Mackay G, Bulusu V, Kamphorst JJ, Tardito S, Strachan D, Harris AL, Aboagye EO, Critchlow SE, Wakelam MJO, Schulze A, Gottlieb E (2015). Acetyl-CoA synthetase 2 promotes acetate utilization and maintains cancer cell growth under metabolic stress. Cancer Cell.

[CR74] Schuler KM, Rambally BS, Difurio MJ, Sampey BP, Gehrig PA, Makowski L, Bae-Jump VL (2015). Antiproliferative and metabolic effects of metformin in a preoperative window clinical trial for endometrial cancer. Cancer Medicine.

[CR75] Song Y, Zhao R, Hu Y, Hao F, Li N, Nie G, Tang H, Wang Y (2015). Assessment of the biological effects of a multifunctional nano-drug-carrier and its encapsulated drugs. Journal of Proteome Research.

[CR76] Southam AD, Khanim FL, Hayden RE, Constantinou JK, Koczula KM, Michell RH, Viant MR, Drayson MT, Bunce CM (2015). Drug redeployment to kill leukemia and lymphoma cells by disrupting SCD1-mediated synthesis of monounsaturated fatty acids. Cancer Research.

[CR77] Southam AD, Payne TG, Cooper HJ, Arvanitis TN, Viant MR (2007). Dynamic range and mass accuracy of wide-scan direct infusion nanoelectrospray Fourier transform ion cyclotron resonance mass spectrometry-based metabolomics increased by the spectral stitching method. Analytical Chemistry.

[CR78] Stäubert C, Bhuiyan H, Lindahl A, Broom OJ, Zhu Y, Islam S, Linnarsson S, Lehtiö J, Nordström A (2015). Rewired Metabolism in Drug-resistant Leukemia Cells a metabolic switch hallmarked by reduced dependence on exogenous glutamine. Journal of Biological Chemistry.

[CR79] St-Coeur P-D, Poitras JJ, Cuperlovic-Culf M, Touaibia M, Morin P (2015). Investigating a signature of temozolomide resistance in GBM cell lines using metabolomics. Journal of Neuro-oncology.

[CR80] Trusheim MR, Berndt ER, Douglas FL (2007). Stratified medicine: strategic and economic implications of combining drugs and clinical biomarkers. Nature Reviews Drug Discovery.

[CR81] van Asten JJ, Vettukattil R, Buckle T, Rottenberg S, van Leeuwen F, Bathen TF, Heerschap A (2015). Increased levels of choline metabolites are an early marker of docetaxel treatment response in BRCA1-mutated mouse mammary tumors: an assessment by ex vivo proton magnetic resonance spectroscopy. Journal of Translational Medicine.

[CR82] Vander Heiden MG, Cantley LC, Thompson CB (2009). Understanding the warburg effect: the metabolic requirements of cell proliferation. Science.

[CR83] Vétizou M, Pitt JM, Daillère R, Lepage P, Waldschmitt N, Flament C, Rusakiewicz S, Routy B, Roberti MP, Duong CP (2015). Anticancer immunotherapy by CTLA-4 blockade relies on the gut microbiota. Science.

[CR84] Wallace DC (2012). Mitochondria and cancer. Nature Reviews Cancer.

[CR85] Wang HJ, Wang L, Zhang HL, Deng PC, Chen J, Zhou B, Hu J, Zou J, Lu WJ, Xiang P, Wu TM, Shao XN, Li Y, Zhou ZG, Zhao YL (2013). H-1 NMR-based metabolic profiling of human rectal cancer tissue. Molecular Cancer.

[CR86] Wang H, Zhao Y, Wu Y, Hu Y-L, Nan K, Nie G, Chen H (2011). Enhanced anti-tumor efficacy by co-delivery of doxorubicin and paclitaxel with amphiphilic methoxy PEG-PLGA copolymer nanoparticles. Biomaterials.

[CR87] Warburg O (1956). Origin of cancer cells. Science.

[CR88] Wise DR, Thompson CB (2010). Glutamine addiction: a new therapeutic target in cancer. Trends in Biochemical Sciences.

[CR89] Wishart DS (2015). Is cancer a genetic disease or a metabolic disease?. Ebiomedicine.

[CR90] Wittmann BM, Stirdivant SM, Mitchell MW, Wulff JE, McDunn JE, Li Z, Dennis-Barrie A, Neri BP, Milburn MV, Lotan Y, Wolfert RL (2014). Bladder cancer biomarker discovery using global metabolomic profiling of urine. PLoS One.

[CR91] Xie GX, Zhou BS, Zhao AH, Qiu YP, Zhao XQ, Garmire L, Shvetsov YB, Yu H, Yen Y, Jia W (2015). Lowered circulating aspartate is a metabolic feature of human breast cancer. Oncotarget.

[CR92] Yang L, Cui XG, Zhang NN, Li M, Bai Y, Han XH, Shi YK, Liu HW (2015). Comprehensive lipid profiling of plasma in patients with benign breast tumor and breast cancer reveals novel biomarkers. Analytical and Bioanalytical Chemistry.

[CR93] Yun J, Mullarky E, Lu C, Bosch KN, Kavalier A, Rivera K, Roper J, Chio IIC, Giannopoulou EG, Rago C, Muley A, Asara JM, Paik J, Elemento O, Chen Z, Pappin DJ, Dow LE, Papadopoulos N, Gross SS, Cantley LC (2015). Vitamin C selectively kills KRAS and BRAF mutant colorectal cancer cells by targeting GAPDH. Science.

[CR94] Zang XL, Jones CM, Long TQ, Monge ME, Zhou MS, Walker LD, Mezencev R, Gray A, McDonald JF, Fernandez FM (2014). Feasibility of detecting prostate cancer by ultraperformance liquid chromatography–mass spectrometry serum metabolomics. Journal of Proteome Research.

[CR95] Zeng J, Huang X, Zhou L, Tan Y, Hu C, Wang X, Niu J, Wang H, Lin X, Yin P (2015). Metabolomics identifies biomarker pattern for early diagnosis of hepatocellular carcinoma: from diethylnitrosamine treated rats to patients. Scientific Reports..

[CR96] Zhu J, Djukovic D, Deng L, Gu H, Himmati F, Zaid MA, Chiorean EG, Raftery D (2015). Targeted serum metabolite profiling and sequential metabolite ratio analysis for colorectal cancer progression monitoring. Analytical and Bioanalytical Chemistry.

